# Iatrogenic Meningitis Caused by *Neisseria sicca/subflava* after Intrathecal Contrast Injection, Australia

**DOI:** 10.3201/eid2006.131117

**Published:** 2014-06

**Authors:** Damoon Entesari-Tatafi, Mohammad Bagherirad, Doreen Quan, Eugene Athan

**Affiliations:** Barwon Health, Geelong, Victoria, Australia

**Keywords:** Iatragenic, bacteria, bacterial meningitis, *Neisseria sicca/subflava*, commensal, lumbar puncture, myelography, intrathecal, facemask, face mask, Australia

## Abstract

We report a case of invasive *Neisseria sicca/subflava* meningitis after a spinal injection procedure during which a face mask was not worn by the proceduralist. The report highlights the importance of awareness of, and adherence to, guidelines for protective face mask use during procedures that require sterile conditions.

*Neisseria sicca/subflava* is a known commensal bacterium of the upper respiratory tract and has rarely been found to cause meningitis, endocarditis, or bacteremia (*1*,*2*). There is limited literature describing the clinical course and optimal management of iatrogenic meningitis caused by *N. sicca/subflava*. Infections of the central nervous system caused by this organism occur rarely; most reported cases are in the pediatric population (*3*,*4*). In the literature describing illness in adults, 4 cases of *N. sicca* meningitis are described, 1 of which was an iatrogenic case: a complication of ventriculostomy (*5*). Of 2 case reports of iatrogenic *N. subflava* meningitis (*6**,**7*) 1 case occurred 48 hours after intrathecal injection in a young immunocompetent female patient (*7*). 

Iatrogenic meningitis is a well-documented complication of lumbar puncture and carries an estimated mortality of ~35% extrapolated from a US data review (*8*). Most cases occur after catheter insertion or injection into the intrathecal space, but infection related to diagnostic lumbar puncture is less common. The most frequently identified causative organisms in samples are *Streptococcus salivarius*, *Streptococcus viridans* and other α-hemolytic streptococci, *Staphylococcus aureus*, and *Pseudomonas* spp. (*9*).

Multiple case reports of iatrogenic meningitis associated with nonuse of face masks prompted a review of the evidence by the Healthcare Infection Control Practices Advisory Committee, which advises US Health and Human Services. The result was a recommendation for the routine use of face masks for clinicians placing a catheter or injecting material into the epidural or spinal space, which was included in the guideline, 2007 Safe Injection Practices to Prevent Transmission of Infection to Patients (*10*). After subsequent outbreaks, the US Centers for Disease Control and Prevention released a clinical reminder in 2011 (*11*).

## The Case

We report the case of an independent 78-year-old man with low back pain and mild lower limb weakness in whom iatrogenic meningitis and associated bacteremia developed after a computed tomography myelogram. The procedure was performed on June 21, 2013, on an outpatient basis in the radiology department of Geelong Hospital, a teaching tertiary hospital. The patient’s medical history included atrial fibrillation that was managed by a permanent pacemaker, which precluded the use of magnetic resonance imaging. Other conditions in his medical history included hypertension, gout, rash after penicillin exposure, and moderate chronic obstructive pulmonary disease that did not require long-term prednisone.

The patient underwent fluoroscopy-guided spinal injection of 10 mL of iohexol 300 mgI/mL contrast medium from a single-dose vial prepared by a nurse in accordance with Centers for Disease Control and Prevention guidelines (*10*). The procedure was performed at the level of lumbar disc space 4–5 by using a 22-gauge spinal needle. Aseptic measures included the use of sterile gloves, gown, drapes, and adequate skin antisepsis by the proceduralist. However, in conflict with hospital policy, a face mask was not used. The procedure was prolonged because the patient’s challenging anatomy required multiple passes. The opening pressure was normal; the patient showed no signs of complications immediately post-procedure and was discharged after 4 hours of observation.

The patient came to the emergency department within 18 hours of the procedure after onset of confusion, severe headache, neck pain, nausea, vomiting, and fever. The patient had a deteriorating conscious state; the examination showed no additional remarkable findings. Specifically, there was no meningism, photophobia, or focal neurologic deficit. The puncture site was not inflamed; no other source of infection was identified.

The patient’s condition was investigated by using a septic screen, including blood cultures and a diagnostic lumbar puncture. Subsequently, treatment with intravenous ceftazidime, vancomycin, and dexamethasone were commenced for presumed iatrogenic meningitis. The initial investigations showed a leukocyte count of 16.1×10^9^/L and a C-reactive protein level of 9.8 mg/L that increased to 185 mg/L within 24 hours of the patient’s return. The diagnostic lumbar puncture revealed turbid cerebrospinal fluid consisting of 4,260 × 10^6^/L leukocytes, 99% polymorphonuclear leukocytes, 270 × 10^6^/L erythrocytes, and levels of 3.0 g/L protein, and 1.7 mmol/L glucose.

Initial microscopic examination of cerebrospinal fluid revealed a gram-positive coccus; on review, intracellular diplococci were identified, suggesting an undercolored specimen that likely represented *Neisseria* species. Ceftazidime was discontinued and intravenous ceftriaxone, 2 g twice daily, was initiated; vancomycin was stopped on confirmation of *N. sicca/subflava* infection. A single set of blood cultures initiated on admission were also positive for this bacterial species, but subsequent blood cultures were negative. The final isolates of *N. Sicca/subflava* from blood culture and cerebrospinal fluid were penicillin resistant but ceftriaxone sensitive.

The patient required 24 hours of management in the intensive care unit because of profound confusion and severe agitation. He improved substantially during a period of 7 days in the hospital and was transitioned from intravenous ceftriaxone to a 7-day course of oral ciprofloxacin, 500 mg twice daily, to be completed after discharge. On discharge, the patient had improved to his baseline level of cognitive function.

The proceduralist who performed the spinal injection denied upper respiratory tract symptoms, but admitted to not using a face mask because of unawareness of the hospital lumbar puncture protocol mandating face mask use when conducting all lumbar punctures. A nasopharyngeal swab specimen confirmed that the proceduralist was a carrier of *N. sicca/subflava* who had an identical antibacterial drug resistance pattern to that identified in the case-patient. Molecular typing of the organism could not be performed because the patient’s isolate had been discarded.

Review by the infection control team identified the low level of awareness of and adherence to the hospital protocol for wearing face masks as a contributory factor. No other cases of iatrogenic meningitis could be traced to the proceduralist.

## Conclusions

We describe the clinical course of iatrogenic meningitis caused by *N. sicca/subflava* with associated bacteremia after a spinal injection procedure. The suspected mechanism of transmission in this case is contamination of the sterile field or equipment by oropharyngeal secretions caused by nonuse of a face mask by a carrier of this organism. The prolonged and technically difficult nature of the procedure likely contributed to contamination by increasing exposure. This hypothesis is supported by the isolation of *N. sicca/subflava* with an identical antimicrobial resistance pattern in a swab sample from the proceduralist’s nasopharynx. An alternative mechanism could involve oropharyngeal secretions from assistant staff or contamination of the contrast medium for spinal injection, although the latter is less likely because it was prepared in accordance with guidelines.

In this case, nonadherence to face mask use standards resulted from lack of awareness by the clinician. To improve clinician awareness after this event, the infection control unit of the hospital updated its lumbar puncture protocol. The protocol mandated the use of face masks for all lumbar punctures, and it was disseminated to all clinical areas where lumbar punctures were performed.

We believe that the best method for promotion of face mask use is making face masks available in preparatory areas and procedure rooms and requiring that all lumbar punctures are performed with the use of a face mask. We also believe there is a need for a system that maintains vigilance. This could include documentation of face mask use for all lumbar punctures and intermittent auditing.

In conclusion, *N. sicca/subflava*, an organism that is harmless in the human oropharynx, can cause invasive infection in immunocompetent adults when introduced directly into the subarachnoid space. Prevention is essential; thus, wearing of face masks should be mandatory for all personnel present during lumbar punctures and all other sterile procedures, and compliance should be monitored.
